# Both the elongation of attached crossbridges and residual force enhancement contribute to joint torque enhancement by the stretch-shortening cycle

**DOI:** 10.1098/rsos.161036

**Published:** 2017-02-15

**Authors:** Atsuki Fukutani, Jun Misaki, Tadao Isaka

**Affiliations:** 1 Faculty of Kinesiology, University of Calgary, 2500 University Drive, NW, Calgary, Alberta, CanadaT2N 1N4; 2 Japan Society for the Promotion of Science, Postdoctoral Fellow for Research Abroad, 5-3-1, Chiyoda-ku, Tokyo 102-0083, Japan; 3 Research Organization of Science and Technology, Ritsumeikan University, 1-1-1 Noji-higashi, Kusatsu, Shiga 525-8577, Japan; 4 Graduate School of Sport and Health Science, Ritsumeikan University, 1-1-1 Noji-higashi, Kusatsu, Shiga 525-8577, Japan; 5 Faculty of Sport and Health Science, Ritsumeikan University, 1-1-1 Noji-higashi, Kusatsu, Shiga 525-8577, Japan

**Keywords:** muscle, crossbridge, titin, elastic energy, eccentric contraction

## Abstract

This study examined the influence of the elongation of attached crossbridges and residual force enhancement on joint torque enhancement by the stretch-shortening cycle (SSC). Electrically evoked submaximal tetanic plantar flexions were adopted. Concentric contractions were evoked in the following three conditions: after 2 s isometric preactivation (ISO condition), after 1 s isometric then 1 s eccentric preactivation (ECC condition), and after 1 s eccentric then 1 s isometric preactivation (TRAN condition). Joint torque and fascicle length were measured during the concentric contraction phase. While no differences in fascicle length were observed among conditions at any time points, joint torque was significantly higher in the ECC than TRAN condition at the onset of concentric contraction. This difference would be caused by the dissipation of the elastic energy stored in the attached crossbridges induced by eccentric preactivation in TRAN condition due to 1 s transition phase. Furthermore, joint torques observed 0.3 and 0.6 s after concentric contraction were significantly larger in the ECC and TRAN conditions than in the ISO condition while no difference was observed between the ECC and TRAN conditions. Since the elastic energy stored in the attached crossbridges would have dissipated over this time frame, this result suggests that residual force enhancement induced by eccentric preactivation also contributes to joint torque enhancement by the SSC.

## 1. Introduction

In almost all human movements, each joint moves dynamically through the actions of concentric and eccentric contractions. Thus, to understand the mechanism of human movements, the force-generating capability of these contractions during complicated tasks should be examined. One of the most interesting themes related to these processes is the stretch-shortening cycle (SSC) [1–3]. Generally, the SSC is comprised of a concentric contraction phase (main contraction) and a preceding eccentric contraction phase (preliminary contraction). In addition, there is an isometric contraction phase (transition period) between the eccentric and concentric contraction phases, which is sometimes called the amortization phase and/or coupling time [4–6]. Because of the preceding eccentric contraction, which is called countermovement [7–9], muscle force/joint torque attained during the concentric contraction phase is increased [10,11].

To understand the mechanism of muscle force/joint torque enhancement by the SSC (i.e. SSC effect), many studies have been conducted *in vivo* [12,13], *in situ* [1,14,15] and *in silico* [16,17]. Taken together, these studies suggest that the SSC effect may be caused by the following factors: the stretch reflex [18,19], tendon elongation [11,13], preactivation [16,20] and residual force enhancement [21,22]. However, there are other studies that have reported that some of these factors may not contribute to the SSC effect [16,23,24]. Further investigation is therefore needed for a clearer understanding of the factors contributing to the SSC effect.

Recently, Fukutani *et al*. [25] suggested that preactivation and residual force enhancement contribute to the SSC effect in plantar flexors in humans. In particular, the authors found that joint torque observed during the concentric contraction phase was larger when isometric preactivation was conducted than when it was not. In addition, a larger SSC effect was observed when preactivation was conducted by eccentric rather than isometric contraction. Since residual force enhancement is evoked by eccentric contraction [21,26], the authors concluded that this factor may also contribute to the SSC effect.

However, these results should be interpreted with caution. In addition to between-group differences in whether or not eccentric contraction was included as preactivation, joint torque at the onset of concentric contraction was also different, i.e. larger in the condition where eccentric rather than isometric preactivation was conducted. This difference in joint torque, which has also been confirmed in the previous study [1], may therefore contribute to a larger SSC effect following eccentric preactivation rather than residual force enhancement.

Regarding this point, the following concept should be useful to solve the above problem. Ruiter *et al*. [27] separated the components that contributed to the joint torque enhancement by active lengthening into a crossbridge-related component and a non-crossbridge-related component. The first component is related to the elongation of attached crossbridges and the second component is related to residual force enhancement [28–30]. Because the former is caused by elongation and rotation of attached crossbridges by active lengthening, that is, eccentric contraction [29,31], this component is dissipated quickly due to the short time frame over which the crossbridge cycle occurs. Specifically, an attached crossbridge is detached from an actin filament, resulting in the dissipation of elastic energy stored in the attached crossbridge. On the other hand, the latter component would be caused by titin–actin interaction and/or increased titin stiffness in the presence of Ca^2+^ [32,33], and consequently, would last several seconds [34,35]. Thus, these components can be distinguished from one other by the time after active lengthening in which they occur, that is, whether they are a short (elongation of attached crossbridges-related) or long-lasting (residual force enhancement-related) component.

Therefore, the purpose of this study was to distinguish the influences of the elongation of attached crossbridges and residual force enhancement on the SSC effect. To this end, the following three conditions were set: (1) concentric contraction just after isometric preactivation, (2) concentric contraction just after eccentric preactivation, (3) concentric contraction after eccentric preactivation then 1 s isometric preactivation, i.e. where a substantial transition period was included between eccentric preactivation and concentric contraction. In condition 3, joint torque enhancement obtained by an elastic energy stored in the attached crossbridges at the onset of concentric contraction should disappear during the transition period. Thus, if joint torque enhancement is caused by the elongation of attached crossbridges only, joint torque during concentric contraction should be similar between conditions 1 and 3. On the other hand, if the influence of residual force enhancement is substantial, joint torque during concentric contraction would be larger in condition 3 than in condition 1.

## 2. Material and methods

### 2.1. Subjects

Twelve healthy young men (age, 24.9 ± 3.8 years; height, 1.73 ± 0.04 m; body mass, 67.5 ± 7.7 kg; all values presented as mean ± s.d.) voluntarily participated in this study.

### 2.2. Experimental set-ups

Similar to a previous study [25], plantar flexion (PF) with a dynamometer (Biodex; SAKAImed, Tokyo, Japan) was adopted as the tested motion. Subjects lay on their back on the dynamometer, and the knee and hip joints were fixed at 0 degrees (i.e. anatomical position). The ankle joint was fixed by the attachment of the dynamometer, with the centres of rotation of the attachment and ankle joint aligned as precisely as possible.

All muscle contractions were evoked by electrical stimulation (SEN-3401; Nihon Kohden, Tokyo, Japan) to rule out the influence of the stretch reflex, which is one of the mechanisms proposed to contribute to the SSC effect [18,19]. Electrical stimulation electrodes were placed on the belly of the triceps surae. Specifically, an anode (4 × 5 cm) was placed on the proximal side of the triceps surae, while a cathode (4 × 5 cm) was placed on the distal side of the soleus. The parameters of electrical stimulation were as follows: pulse frequency, 50–100 Hz which was sufficient to evoke a fully fused contraction; pulse duration, 0.5 ms; and train duration, 4.0 s. To determine the intensity of electrical stimulation to be used, maximal voluntary isometric contraction in PF was performed with the ankle joint angle at PF0° (anatomical position). The peak joint torque recorded in this contraction was set as 100% intensity. The intensity of electrical stimulation was adjusted to evoke 25% intensity at the identical ankle joint angle. This electrical stimulation intensity was applied to all contractions. Joint torque and joint angle were recorded with a sampling frequency of 4000 Hz (Power lab 16/30; ADInstruments, Bella Vista, Australia).

### 2.3. Motion control of the dynamometer and settings of electrical stimulation

**Figure 1.**
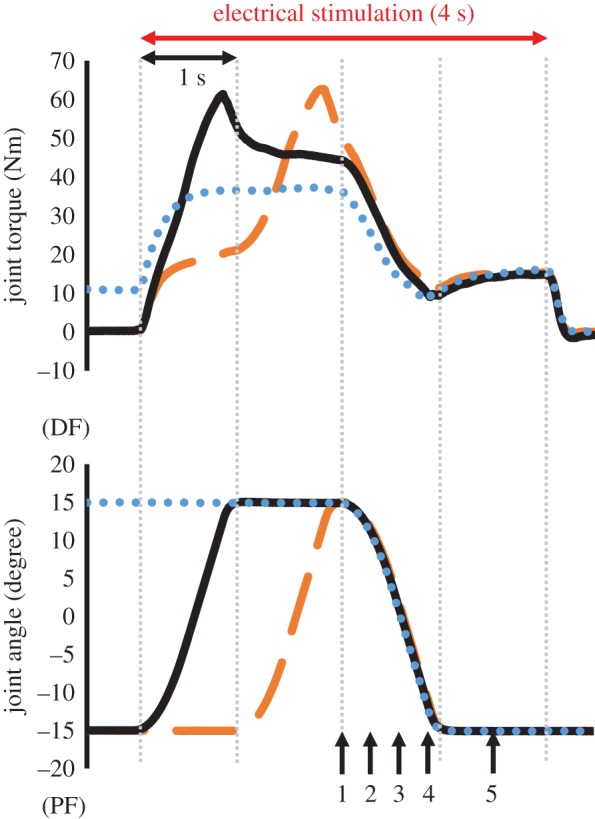
Typical time courses of joint torque and joint angle changes in the three conditions tested (blue dotted line, ISO condition; orange dashed line, ECC condition; black solid line, TRAN condition). The time points at which joint torque, fascicle length and pennation angle were measured (T1–T5) are shown by black arrows.

Three conditions were tested in which the following was carried out: (1) a 1 s concentric contraction was conducted immediately after 2 s isometric preactivation (ISO condition hereafter), (2) a 1 s concentric contraction was conducted after 1 s isometric then 1 s eccentric preactivation (ECC condition hereafter) and (3) a 1 s concentric contraction was conducted after 1 s eccentric then 1 s isometric preactivation so that there was a transition period (i.e. an isometric contraction phase) between eccentric and concentric contractions (TRAN condition hereafter; figure 1). The range of motion of the ankle joint was from PF15° to dorsiflexion 15°. Joint angular velocities of the eccentric and concentric contractions were set at 45° s^−1^. Owing to the acceleration and deceleration phase of the attachment movements, the duration of the eccentric and concentric contractions was 1 s.

In the ISO condition, muscle contraction was started at DF15° in an isometric contraction state. After 2 s passed, the ankle joint was moved to PF15° with a shortening duration of 1 s. Then, the ankle joint angle was held at PF15° for 1 s before stimulation was stopped. In the ECC condition, muscle contraction was started at the PF15° in an isometric contraction state. After 1 s passed, the ankle joint was moved to DF15° with a 1 s lengthening duration. Immediately after reaching DF15°, the ankle joint was moved to PF15° with a shortening duration of 1 s. The ankle joint angle was held at PF15° for 1 s, and then the stimulation was stopped. Finally, in the TRAN condition, muscle contraction was started at PF15°. Immediately after the onset of stimulation, the ankle joint was moved to DF15° with a lengthening duration of 1 s. Then, the joint angle was held at DF15° for 1 s (i.e. the transition period). After 1 s passed, the joint was moved to PF15° with a 1 s shortening duration. The ankle joint angle was then held at PF15° for 1 s before stimulation was stopped.

### 2.4. Ultrasonographic measurement

Ultrasonographic measurement (SSD-3500; Aloka, Tokyo, Japan) was performed simultaneously during the above three trials to examine whether changes in architectural characteristics (i.e. fascicle length and pennation angle) were related to the SSC effect. A linear array probe (7.5 MHz, field of view: 6 × 6 cm, UST-5710; Aloka, Tokyo, Japan) was used to obtain images of the belly of the medial gastrocnemius muscle. The ultrasonographic probe was fixed onto the skin by using underwrap and surgical tape. Fascicle length was defined as the distance between the intersection of the superficial aponeurosis and fascicle and the intersection of the deep aponeurosis and fascicle, while pennation angle was defined as the acute angle formed by the fascicle and deep aponeurosis. Sampling frequency of ultrasonography was 30 Hz. Synchronization of joint torque and joint angle was done by inserting a pulse into the ultrasonographic recording machine. Acquired images were analysed by ImageJ 1.47v software (National Institute of Health, Bethesda, MD, USA).

### 2.5. Analyses and measurements

**Figure 2.**
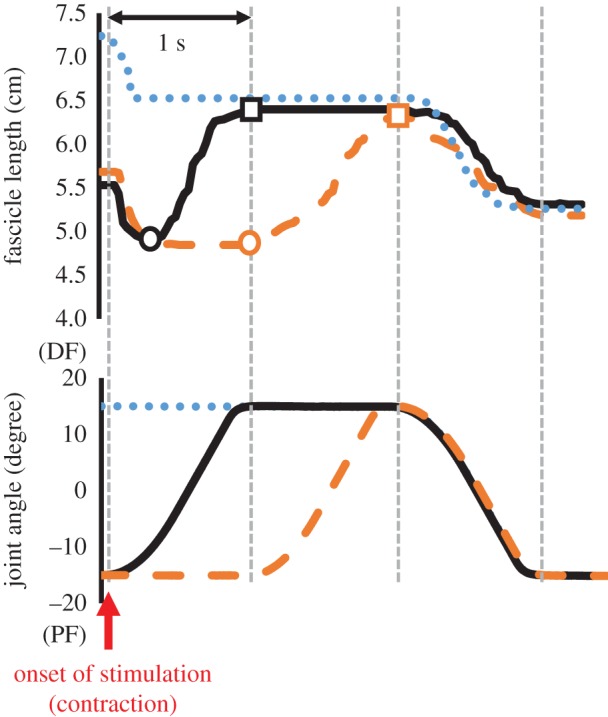
Typical time courses of fascicle length and joint angle changes in the three conditions tested (blue dotted line, ISO condition; orange dashed line, ECC condition; black solid line, TRAN condition). Circles indicate the timing at which fascicle length was the shortest during the eccentric contraction, and squares indicate the timing at which fascicle length was the longest (i.e. end of the eccentric contraction). Note that fascicle length shortened in the initial phase of contractions in all conditions, and fascicle was elongated during eccentric contraction phase in ECC and TRAN conditions.

Because we focused on the joint torque attained during the concentric contraction phase, we compared the joint torques recorded at the following five points: at the onset of concentric contraction (T1), 300 ms after the onset of concentric contraction (T2), 600 ms after the onset of concentric contraction (T3), 900 ms after the onset of concentric contraction (T4), and 1400 ms after the onset of concentric contraction (T5) (see figure 1, black arrows). Fascicle length and pennation angle were also measured at these time points. In addition, to confirm whether elongation of the fascicle occurred in TRAN and ECC conditions, the magnitude of elongation of the fascicle was examined by subtracting the fascicle length at the onset of stimulation from that at the end of eccentric contraction. Furthermore, because the fascicle shortened after inserting the stimulation in TRAN and ECC conditions (figure 2), the shortest fascicle length observed after inserting stimulation was subtracted from the fascicle length at the end of eccentric contraction to calculate the actual elongation of the fascicle.

In our previous study [25], which adopted similar experimental settings, coefficients of variation for joint torque, fascicle length and pennation angle were evaluated. Coefficients of variation for these were 2.3%, 1.1% and 1.6%, respectively, and their intraclass correlations were 0.995, 0.993 and 0.989, respectively.

### 2.6. Statistics

A two-way analysis of variance (ANOVA) with repeated measures was adopted to examine the interaction and main effect of condition and time on joint torque, fascicle length and pennation angle. If the interaction was significant, a one-way ANOVA with repeated measures followed by a *post hoc* test (Bonferroni's correction) was conducted. Effect size for ANOVA was calculated as the partial *η*^2^. To confirm whether the fascicle was elongated or not during the eccentric contraction phase, and to compare the magnitude of elongation of the fascicle between TRAN and ECC conditions, paired *t*-test was conducted. Statistical analyses were performed using SPSS version 20 software (IBM, Tokyo, Japan), with the level of statistical significance set at *p* < 0.05.

## 3. Results

**Figure 3.**
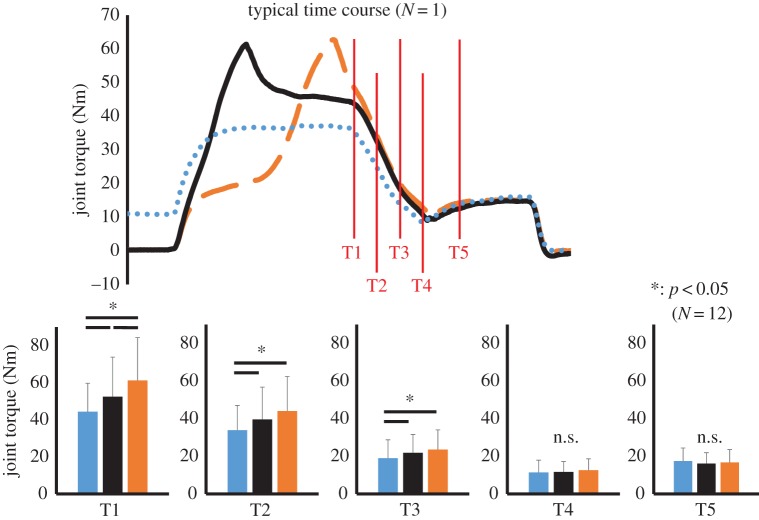
Joint torque observed at the following five points: the onset of concentric contraction (T1), and 300, 600, 900 and 1400 ms after the onset of concentric contraction (T2, T3, T4 and T5, respectively). The blue dotted line and bar show joint torque in the ISO condition, the orange dashed line and bar show joint torque in the ECC condition, and the black solid line and bar show joint torque in the TRAN condition. Asterisk indicates a significant difference between conditions (*p* < 0.05).

For joint torque, a two-way ANOVA with repeated measures revealed a significant interaction between condition and joint angle (*F* = 16.505, partial *η*^2^ = 0.600, *p* < 0.001). Subsequent analyses showed that force was significantly larger in the order of ECC, TRAN and ISO conditions at T1 (*p* = 0.001–0.028). At T2 and T3, joint torque was larger in the ECC and TRAN conditions than in the ISO condition (*p* = 0.001–0.042), but was not different between ECC and TRAN (*p* = 0.131 for T2 and *p* = 0.378 for T3). No differences were observed among conditions at T4 and T5 (*p* = 0.169–0.999; figure 3).

**Figure 4.**
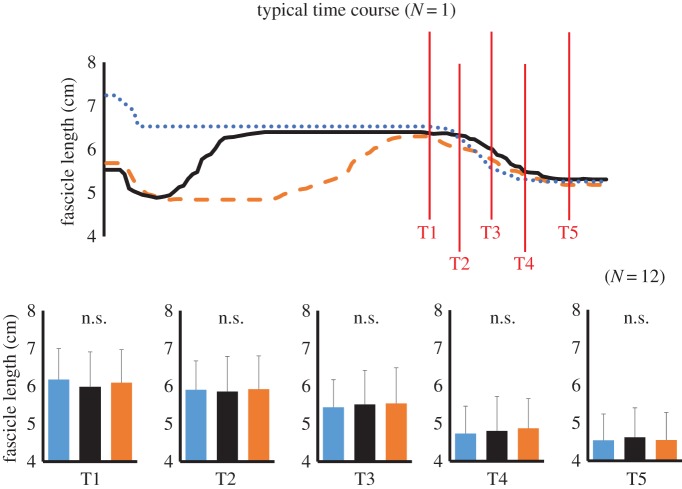
Fascicle length observed at the following five points: the onset of concentric contraction (T1), and 300, 600, 900 and 1400 ms after the onset of concentric contraction (T2, T3, T4 and T5, respectively). The blue dotted line and bar show joint torque in the ISO condition, the orange dashed line and bar show joint torque in the ECC condition and the black solid line and bar show joint torque in the TRAN condition.

Although a significant interaction was found for fascicle length and pennation angle (fascicle length: *F* = 2.656, partial *η*^2^ = 0.195, *p* = 0.012; pennation angle: *F* = 2.160, partial *η*^2^ = 0.164, *p* = 0.038), subsequent analyses showed that no significant difference was observed among conditions for these variables (figure 4 and table 1).

**Table 1.** Pennation angle measured at the onset of concentric contraction (T1), and 300, 600, 900 and 1400 ms after the onset of concentric contraction (T2, T3, T4 and T5, respectively). No significant differences (*p* < 0.05) among conditions.

pennation angle (degree)					
	Point 1	Point 2	Point 3	Point 4	Point 5
ISO	21.7 ± 3.1	22.8 ± 3.7	24.5 ± 3.8	28.3 ± 4.6	29.5 ± 4.2
TRAN	22.6 ± 2.5	23.1 ± 2.8	24.3 ± 3.1	27.7 ± 3.7	28.9 ± 3.1
ECC	22.1 ± 2.4	22.8 ± 2.4	24.0 ± 2.6	27.0 ± 3.0	29.1 ± 2.6

**Figure 5.**
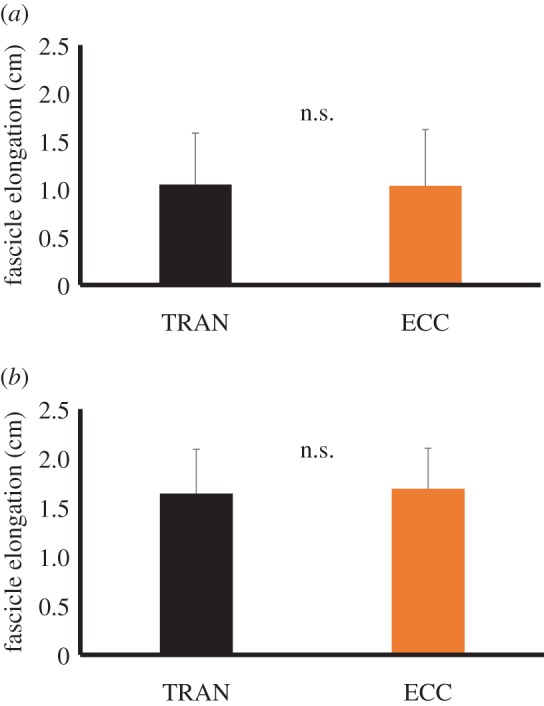
Magnitude of the fascicle elongation in TRAN and ECC conditions. (*a*) The magnitude of fascicle elongation calculated by subtracting the fascicle length before stretch from that at the end of eccentric contraction. (*b*) The magnitude of fascicle elongation calculated by subtracting the shortest fascicle length observed during the eccentric contraction phase from the fascicle length at the end of eccentric contraction (figure 2).

The magnitude of elongation of the fascicle calculated by subtracting the fascicle length at the onset of stimulation from that at the end of eccentric contraction was not different between TRAN (1.0 ± 0.5 cm) and ECC (1.0 ± 0.6 cm) conditions (*p* = 0.901). Furthermore, the magnitude of elongation of the fascicle calculated by subtracting the shortest fascicle length observed after inserting stimulation from the fascicle length at the end of eccentric contraction was not different between TRAN (1.6 ± 0.5 cm) and ECC (1.7 ± 0.4 cm) conditions (*p* = 0.564; figure 5).

## 4. Discussion

The primary purpose of this study was to distinguish between the influence of the elongation of attached crossbridges and residual force enhancement on joint torque enhancement by the SSC. A significant difference in joint torque was found between the ECC and TRAN conditions at the onset of concentric contraction even though a similar magnitude of active lengthening, which determines the magnitude of residual force enhancement [21,36], was included in preactivation. This difference should be caused by an elongation of attached crossbridges induced by the eccentric contraction conducted just before the concentric contraction. Although the extent of elongation of attached crossbridges (i.e. elastic energy stored in the attached crossbridges) at the end of eccentric contraction should be the same between ECC and TRAN conditions, elastic energy stored in the attached crossbridges induced by eccentric contraction in TRAN condition disappeared due to the transition period between eccentric and concentric contraction. On the other hand, it was observed in this study that joint torque attained during the latter phase of concentric contraction (300 and 600 ms after the onset of concentric contraction) was still significantly larger in the ECC and TRAN conditions than in the ISO condition, despite the elastic energy stored in the attached crossbridges already having dissipated. Thus, a mechanism other than elongation of attached crossbridges should contribute to the joint torque enhancement by SSC. This mechanism would be residual force enhancement.

Joint torque at the onset of concentric contraction was larger in the ECC than in the TRAN conditions. This would be caused by elastic energy stored in the attached crossbridges [29,31]. In the ECC condition, concentric contraction was commenced ‘immediately after’ the eccentric contraction. During eccentric contraction, attached crossbridges are elongated so that the magnitude of force per attached crossbridge is increased due to an increase in X-distance (i.e. distance from the equilibrium position) [37,38]. This causes larger joint torque at the onset of concentric contraction. However, this effect should be quickly dissipated because elastic energy stored in the attached crossbridges is released due to the detachment of crossbridges from actin filaments. The duration of the attached state would be at most 200–300 ms [39–41]. Indeed, the difference in joint torque between the ECC and TRAN conditions, which appears to be attributable to the elastic energy stored in the attached crossbridges, disappeared within the early part of concentric contraction (within 300 ms) in this study. This result is in line with previous studies demonstrating that joint torque/force/power enhancement by active lengthening was dissipated within 300 ms [42,43]. Thus, it is reasonable to consider that the elastic energy stored in the attached crossbridges contributes to force enhancement by the SSC, especially during the early phase of concentric contraction.

On the other hand, joint torque enhancement observed in the latter phase (300 ms (T2) and 600 ms (T3) after the onset of concentric contraction) in the ECC and TRAN conditions than in ISO condition cannot be explained by the elastic energy stored in the attached crossbridges, because the influence of the elastic energy stored in the attached crossbridges should have disappeared in the early phase of concentric contraction as described above. In addition, Bosco *et al*. [2] discussed in their manuscript that it was somewhat surprising that large mechanical work by active lengthening was observed despite a long transition period (coupling time) of about 150 ms between eccentric and concentric contractions. This unexplained joint torque enhancement is likely to have instead been caused by residual force enhancement. Residual force enhancement is defined as the increase in muscle force (joint torque) induced by active lengthening [21,22]. Although the definition of residual force enhancement is that the force attained during ‘isometric contraction’ after active lengthening is higher than the force attained during isometric contraction without preliminary active lengthening at the same muscle length [21,26,44], this phenomenon should be applicable even for force attained during ‘concentric contraction’ after active lengthening. This is because residual force enhancement occurs through titin–actin interactions and/or increased titin stiffness [32,33], and increased force through this mechanism should affect not only isometric contractions but also concentric contractions. Therefore, it is reasonable to consider that residual force enhancement would be involved in joint torque enhancement by the SSC [1,25,45].

In the current experiment, the isometric joint torque after eccentric contraction (i.e. transition period) in the TRAN condition was larger than the isometric joint torque at the same joint angle in the ISO condition (figure 1). Therefore, it is reasonable to consider that substantial residual force enhancement occurred in the TRAN condition, and that same amount of residual force enhancement occurred in the ECC condition because the same magnitude of active lengthening was included in the TRAN and ECC conditions. Because residual force enhancement lasts for several seconds [34,35], this effect can explain the joint torque enhancement observed in the latter phase of the concentric contraction in the ECC and TRAN compared to the ISO condition. On the other hand, joint torque enhancement disappeared by 900 ms (T4) and 1400 ms (T5) after the onset of concentric contraction. Because residual force enhancement should be caused by enhanced titin stiffness and should continue several seconds [32–35], it may be reasonable to expect that joint torque enhancement (SSC effect) would continue even in the T4 and T5. Possible explanations about this unexpected result are interaction with residual force depression [28,46] and short muscle length (plantar flexed position) at T4 and T5. First, residual force depression induced by active shortening (negative effect) can mask the effect of residual force enhancement (positive effect). For example, previous studies conducted active shortening after active lengthening (i.e. SSC), and reported similar results; residual force enhancement disappeared after active shortening [47,48]. Second, short muscle fibre length can also explain the observed result. Because muscle fibre length should be short at T4 and T5, in other words, muscle should operate in the ascending limb in this phase, the influence of residual force enhancement should become small due to small or negligible elongation of titin. This can explain the no-SSC effect at T4 and T5. The latter point can be clarified by conducting a similar experiment adopting much longer muscle length even after the end of active shortening, because this experiment can emphasize the influence of residual force enhancement (i.e. titin stiffness).

Although the same amount of joint angle changes (i.e. muscle–tendon complex elongation) was imposed during the eccentric contraction phase in TRAN and ECC conditions, there is a possibility that magnitude of fascicle elongation was different due to tendon elongation. Regarding this point, the magnitude of fascicle elongation was confirmed by ultrasonographic data. As a result, fascicle elongation did occur during the eccentric contraction phase in both conditions, and the magnitude of fascicle elongation was similar between TRAN and ECC conditions. Thus, it is reasonable to assume that elongation of attached crossbridges and residual force enhancement induced by fascicle elongation occurred in our experimental settings.

It is widely considered that tendon elongation is the primary mechanism of the SSC effect [12,13,49]. According to the widely accepted method of evaluating tendon length changes, tendon length change can be estimated by fascicle length changes corrected by pennation angle and joint angle changes [13,50,51]. Considering the fact that joint angle change during the concentric contraction phase was identical among conditions and that no significant differences were observed in fascicle length and pennation angle among conditions (table 1) in our study, tendon length change was also considered to be the same among conditions. Therefore, the difference in joint torque observed among conditions in this study cannot be explained by elastic energy stored in the tendon [52,53] and/or muscle–tendon interaction [54,55]. The negligible contribution of tendon elongation may have been due to weak contraction intensity, which corresponded to 25% of the intensity of maximal voluntary isometric contraction at PF0°. This study adopted submaximal electrically evoked contractions instead of maximal electrically evoked contractions due to intolerable pain caused by the latter, and instead of maximal and/or submaximal voluntary contractions to avoid complicated neural modulations that affect joint torque [56–58]. As a result of adopting relatively low intensity, the magnitude of joint torque difference was not so large among condition (e.g. the largest difference observed at the T1 was about 10 Nm). This joint torque difference should not be enough to induce substantial tendon elongation. Although further studies are needed to clarify the influence of tendon elongation on the SSC effect in higher intensity contraction conditions, we can say based on the current data that differences in joint torque among current experimental conditions were not caused by tendon elongation and/or muscle architecture.

A limitation of this study is that we measured fascicle length from the medial gastrocnemius only. Previous studies reported that medial gastrocnemius and soleus behaved differentially [59]. Thus, there is a possibility that behaviour of soleus and lateral gastrocnemius which contribute to the PF torque made the interpretation more complicated. However, although the magnitude of length changes of these three muscles at a given joint angle changes may be different, the behaviours of these three muscles should be the same between TRAN and ECC conditions. Therefore, this factor would not affect our main result.

When considering the practical application of the current results, the influence of fibre-type dependence on the relative contribution of the elongation of attached crossbridges and residual force enhancement to the SSC effect should be mentioned. Regarding the elongation of attached crossbridges, the duration of the attached state (ATPase rate) is shorter in fast-twitch than slow-twitch fibres [60,61]. Thus, elastic energy stored in the attached crossbridge by active lengthening is released more quickly in fast-twitch fibres. Consequently, the duration over which the elastic energy stored in the attached crossbridges contributes to joint torque enhancement should be shorter in fast-twitch than in slow-twitch fibres. In addition, residual force enhancement, which is caused by titin elasticity [32,33], should also be affected by fibre type because the titin isoform found in slow-twitch fibres is longer than the isoform found in fast-twitch fibres [62,63]. Specifically, the shorter titin isoform produces a larger force at a shorter sarcomere length (muscle length) compared with the longer titin isoform. Thus, even in the same absolute muscle length (joint angle), the influence of residual force enhancement should vary among humans depending on which fibre type is more abundant [64]. Taking these into account, the influence of fibre type on the relative contribution of the elongation of attached crossbridges and residual force enhancement on the SSC effect should be examined in the future.

## 5. Conclusion

In conclusion, it was found that joint torque was larger in the ECC than in the TRAN condition despite both conditions involving the same magnitude of active lengthening. Thus, the joint torque enhancement observed in these conditions can be attributable to the elongation of attached crossbridges. In addition, the joint torque enhancement seen in these conditions during the latter phase of concentric contraction, after the influence of the elongation of attached crossbridges had dissipated, points to the involvement of residual force enhancement. Therefore, both the elongation of attached crossbridges and residual force enhancement contribute to joint torque enhancement by the SSC.

## Supplementary Material

Individual values of all data (joint torque, fascicle length and pennation angle) are shown in the electronic supplementary material.

## Data accessibility

From the view of the protection of personal information, data accessibility is partly restricted by the ethical committee. Individual values of the joint torque, fascicle length and pennation angle were provided in the electronic supplementary material while individual values of year, height and body mass were not provided.
